# Supplementary material for: Ultra-Wideband Radar for Simultaneous and Unobtrusive Monitoring of Respiratory and Heart Rates in Early Childhood: A Deep Transfer Learning Approach
Source: Sensors (Basel). 2023 Sep 5;23(18):7665. doi: 10.3390/s23187665 (PMC10535330; doi:10.3390/s23187665)
Supplement: Supplementary file 1 [file sensors-23-07665-s001.zip › sensors-2539517-supplementary.pdf]

To compare the whole 293 hours of synchronized video and radar data, we computed moving standard deviation of video pixels (showing change and movement in the frames) and movement of radar data for the corresponding 14204821 frames(for down-sampled video data). Fig S1 depicts the normalized values of movement and pixel intensity while showing horizontal line of threshold (300/max(movement)). It is obvious that the threshold value of 300 (normalized to 0.0147), although maybe too strict, is lower than the majority of simultaneous sharp rises in video intensity change and radar movement.

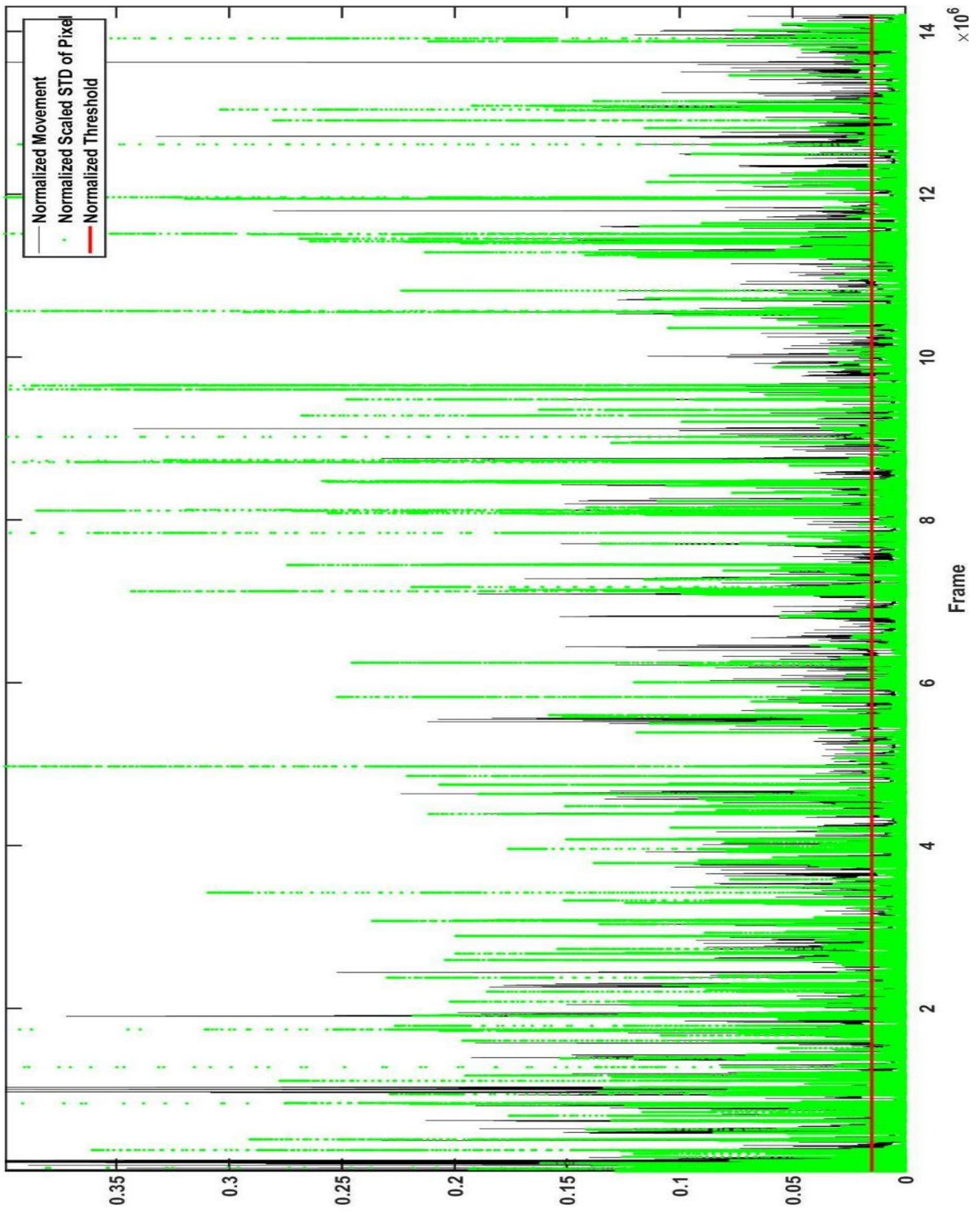

Figure S1. normalized values of movement and pixel intensity

Figure S2 demonstrates how the spectrograms of the signal for the bin (related to corresponding range) with the biggest power of frequency component of radar down-converted could be different for different magnitudes of HR and RR. It is obvious that the time frequency components of received signal are directly related to the respiratory and heartbeat of moving target chest-wall. One interesting results is the way that in state of maximum BR (HR), received radar signals are coded differently when the HR (BR) is not maximum.

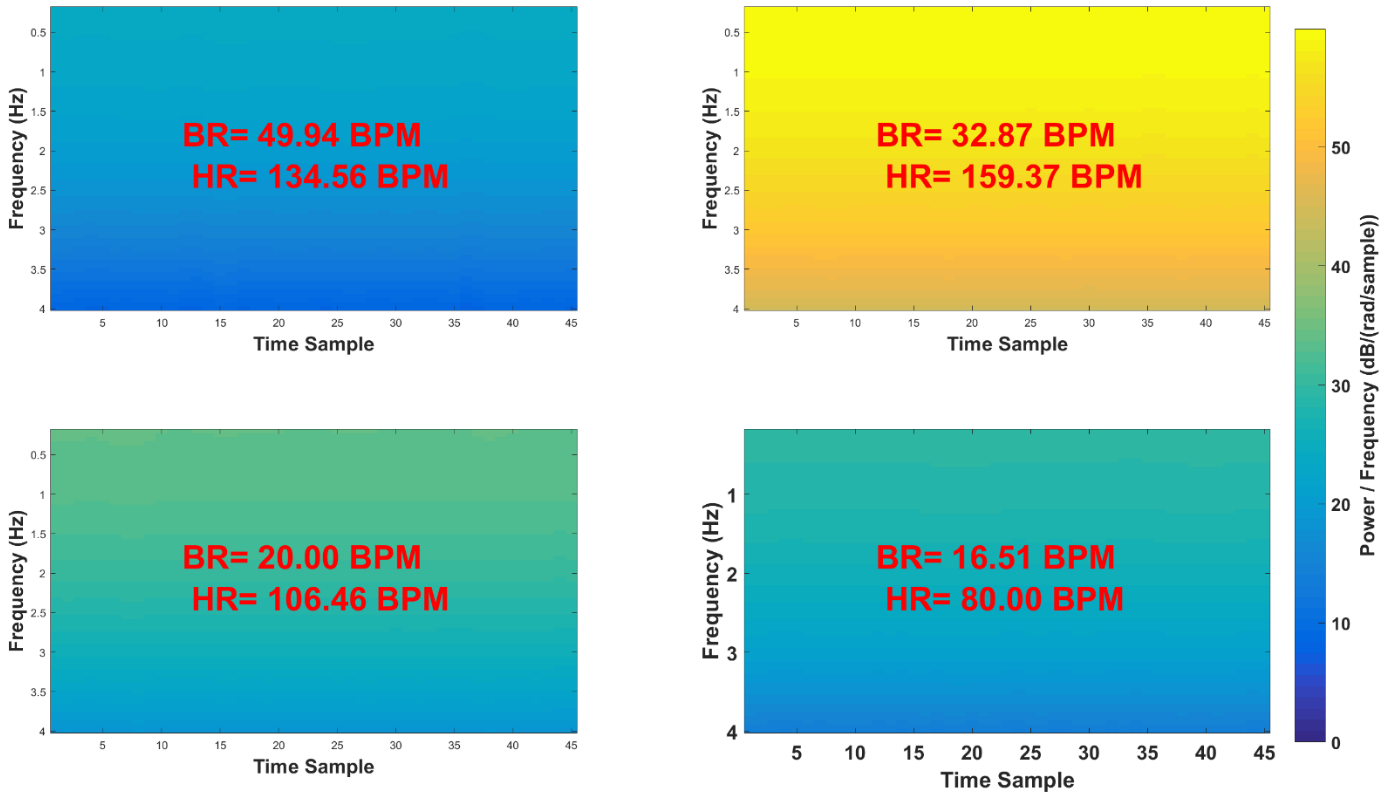

Figure S2. Different spectrogram values for 4 different sets of HR/BR magnitudes.
